# Stability of infraslow correlation structure in time-shifted intracranial EEG signals

**DOI:** 10.3389/fnetp.2024.1441294

**Published:** 2024-08-27

**Authors:** Rasesh B. Joshi, Robert B. Duckrow, Irina I. Goncharova, Lawrence J. Hirsch, Dennis D. Spencer, Dwayne W. Godwin, Hitten P. Zaveri

**Affiliations:** ^1^ Department of Neurology, Boston Children’s Hospital, Harvard Medical School, Boston, MA, United States; ^2^ Department of Translational Neuroscience, Wake Forest University School of Medicine, Winston-Salem, NC, United States; ^3^ Yale Clinical Neuroscience Neuroanalytics, Yale University, New Haven, CT, United States; ^4^ Department of Neurology, Yale University, New Haven, CT, United States; ^5^ Department of Neurosurgery, Yale University, New Haven, CT, United States

**Keywords:** epilepsy, intracranial EEG, magnitude squared coherence (MSC, ), infraslow activity, EEG

## Abstract

It is increasingly understood that the epilepsies are characterized by network pathology that can span multiple spatial and temporal scales. Recent work indicates that infraslow (<0.2 Hz) envelope correlations may form a basis for distant spatial coupling in the brain. We speculated that infraslow correlation structure may be preserved even with some time lag between signals. To this end, we studied intracranial EEG (icEEG) data collected from 22 medically refractory epilepsy patients. For each patient, we selected hour-long background, awake icEEG epochs before and after antiseizure medication (ASM) taper. For each epoch, we selected 5,000 random electrode contact pairs and estimated magnitude-squared coherence (MSC) below 0.15 Hz of band power time-series in the traditional EEG frequency bands. Using these same contact pairs, we shifted one signal of the pair by random durations in 15-s increments between 0 and 300 s. We aggregated these data across all patients to determine how infraslow MSC varies with duration of lag. We further examined the effect of ASM taper on infraslow correlation structure. We also used surrogate data to empirically characterize MSC estimator and to set optimal parameters for estimation specifically for the study of infraslow activity. Our empirical analysis of the MSC estimator showed that hour-long segments with MSC computed using 3-min windows with 50% overlap was sufficient to capture infraslow envelope correlations while minimizing estimator bias and variance. The mean MSC decreased monotonically with increasing time lag until 105 s of lag, then plateaued between 106 and 300 s. Significantly nonzero infraslow envelope MSC was preserved in all frequency bands until about 1 min of time lag, both pre- and post-ASM taper. We also saw a slight, but significant increase in infraslow MSC post-ASM taper, consistent with prior work. These results provide evidence for the feasibility of examining infraslow activity via its modulation of higher-frequency activity in the absence of DC-coupled recordings. The use of surrogate data also provides a general methodology for benchmarking measures used in network neuroscience studies. Finally, our study points to the clinical relevance of infraslow activity in assessing seizure risk.

## 1 Introduction

The epilepsies are a family of disorders characterized by a propensity toward spontaneous, recurrent seizures. Whereas seizures were traditionally thought to represent an imbalance between excitation and inhibition in circumscribed foci in the brain, it is now increasingly evident that in many patients, these pathological states likely arise from evolving networks. The notion of an epilepsy network was first put forth in the seminal paper by Spencer (2002). In the 2 decades since, this network conceptualization has proven essential in both our mechanistic and clinical understanding of the epilepsies. Coupled with this, there is also a growing understanding of how interacting multiscale rhythms play a role both in normal physiology and in pathology (Buzsáki and Vöröslakos, 2023). Specifically in epilepsy, seizure risk seems to be modulated by rhythms across a wide temporal range from high-frequency oscillations to circadian and multidien cycles (Baud et al., 2018). Of these many rhythms, infraslow oscillations have been of recent interest.

Infraslow oscillations (ISOs), which are typically defined as low-frequency activity below 0.2 Hz, have been described for many decades (Aladjalova, 1957; Aladjalova, 1964). A recent resurgence of interest in ISOs was primarily due to their potential correspondence with blood-oxygen-level dependent (BOLD) fluctuations as measured by MRI. We previously reported a lack of correspondence between the fMRI-defined default mode network (DMN) and infraslow envelope correlations (i.e., the second spectrum) of the intracranial EEG (icEEG) (Joshi et al., 2016). However, our results and a number of studies using other modalities such as magnetoencephalography indicate that envelope correlations in the infraslow range might form a basis for distant spatial coupling in the brain (Hipp et al., 2012; Nir et al., 2008). Briefly, in prior analysis of resting data, we found that infraslow envelope relationship decreased with greater intercontact distance, relationships were strongest in the delta band, and that they decreased with increasing frequency (Joshi et al., 2016). Further, ISOs appear to play a role in modulating faster frequencies as evidenced by phase-amplitude coupling with higher-frequency activity (Vayrynen et al., 2023). These measures also appear to have utility in understanding pathological brain states, particularly seizures and epilepsy. We previously found that changes in infraslow envelope correlation structure consistently occur in periods when patients are more vulnerable to seizure (Joshi et al., 2018).

Given that infraslow activity includes a wide range of frequencies that span a timescale of many seconds to minutes, it is important to better characterize the exact activity that is captured in studies of infraslow functional connectivity. Initial studies of infraslow activity have suggested that the slowest activity observed in this range possessed periods of up to 2 min (Aladjalova, 1964). Though network studies of infraslow activity have provided significant insight, the functional connectivity measures used in these studies have generally not been thoroughly characterized. Importantly, the study of infraslow activity necessitates segments of data that are sufficiently long to capture ISOs possessing periods on the scale of minutes, an issue that is often overlooked. Estimation methods that rely on further segmentation and windowing of data, such as the Welch’s overlapped segmentation approach (WOSA) for magnitude-squared coherence (MSC) and others, must also be optimized to use windows that are sufficiently large to capture low-frequency activity, while still minimizing estimator bias and variance.

To this end, there were three main objectives for our study. First, we used surrogate data to empirically characterize the MSC estimator as applied specifically to the study of infraslow activity in the intracranial EEG and to provide parameters for optimal estimation. This use of surrogate data also provides a framework for methodology that can be used to benchmark network measures in neuroscience. Second, as the slow modulations of interest in our study contain activity on the order of many seconds to minutes, we sought to determine whether correlation structure may be preserved even with some time lag between signals. We therefore used time lag analysis to study the timescale of these infraslow envelope correlations. Finally, we examined how correlation structure changes over the period of icEEG monitoring, specifically during periods before and after antiseizure medication (ASM) taper.

## 2 Methods

### 2.1 Patient selection

We selected and studied icEEG data from 22 medically refractory adult epilepsy patients (aged 18 and older) who underwent surgical evaluation and seizure onset zone (SOZ) localization at Yale-New Haven Hospital. Of these, 10 patients were female and 12 were male, and they had a mean age of 32.3 years. Further information about individual patients may be found in Table 1. The patients provided written informed consent for analyses of their records.

**TABLE 1:** Patient information.

Patient	Sex	Age	Seizure onset zone	Antiseizure medications
1	M	54	Bilateral anterior hippocampi	CBZ, ZNS
2	F	27	Right medial anterior temporal	LEV, TPM
3	M	36	Not localized	CBZ, PGB
4	M	31	Right inferior medial temporal extending posteriorly	LEV, OXC
5	M	54	Right inferior temporal	LEV, OXC
6	M	27	Anterior lateral temporal	CBZ, LTG
7	M	51	Left medial temporal	GBP, LEV, OXC, VPA
8	F	39	Left medial temporal	CBZ, VPA, ZNS
9	M	35	Left inferior parietal-occipital	PHT, VPA, ZNS
10	M	27	Left medial temporal	OXC, PHT
11	F	19	Right occipital pole	OXC, ZNS
12	F	31	Right inferior posterior temporal-occipital	LEV, OXC
13	F	41	Right anterior lateral parietal	CBZ, LTG, PBB
14	F	24	Left medial occipital	CBZ, GBP
15	F	35	Left superior parietal lobule	CBZ, PHT, CLN
16	F	26	Right posterior medial frontal-parietal	CBZ, TPM
17	F	18	Right anterior and inferior temporal	LEV, OXC
18	M	26	Left medial temporal	PGB, PHT, ZNS
19	M	20	Left anterior and medial frontal	CBZ, LEV, ZNS
20	M	38	Left medial temporal and inferior temporal	LTG, OXC
21	M	23	Right temporal-parietal-occipital	CBZ, LTG, TPM
22	F	28	Right medial temporal	LEV, PHT, ZNS

CBZ, carbamazepine; CLN, clonazepam; ZNS, zonisamide; LEV, levetiracetam; TPM, topiramate; PGB, pregabalin; OXC, oxcarbazepine; VPA, valproate; LTG, lamotrigine; PBB, phenobarbital; PHT, phenytoin.

### 2.2 Intracranial EEG acquisition and selection of epochs

Intracranial macro depth, strip, and grid electrodes (Ad-Tech Medical Instrument Corporation, Racine, WI) were implanted based on an approach uniquely tailored to each individual patient. The icEEG and simultaneous video were recorded and sampled at 256 Hz with a commercially available 128-channel long-term video-icEEG monitoring system (Natus/Bio-logic Systems Incorporated, San Carlos, CA). The reference used for these recordings was a peg electrode implanted in the diploic space of the skull at a distance from all icEEG electrodes. We used the referential recording for all analyses.

We sought to determine whether long-term correlation structure is preserved in time-shifted resting icEEG signals. In a prior study of a smaller cohort of patients, we found that infraslow envelope MSC increases after ASM taper (Joshi et al., 2018). As part of icEEG monitoring, patients are typically tapered off their antiseizure medications (ASMs) to elicit a greater number of seizures that can be used for SOZ localization. We therefore chose to study resting data both prior to and after ASM taper. We selected an hour-long background icEEG segment before and after ASM taper for each patient. The selected epochs were time periods when patients appeared to be resting quietly with eyes open as determined retrospectively by the video and icEEG record (i.e., patients were not directed to be in a non-task state). To minimize the possibility of contamination of these data by seizure or potential pre-seizure changes, the selected epochs were at least 6 h removed from seizure.

### 2.3 Characterization of the infraslow envelope magnitude-squared coherence estimator

We computed measures of infraslow envelope MSC as reported previously (Joshi et al., 2016; Joshi et al., 2018). Briefly, we computed running power in the traditional EEG frequency bands (delta [0.5–4 Hz], theta [4–8 Hz], alpha [8–13 Hz], beta [13–25 Hz], and gamma [25–55 Hz]) at a 1-s resolution over each of the hour-long icEEG epochs. To quantify correlations in infraslow amplitude modulations of these band power time-series, we estimated MSC between all possible electrode contact pairs for each 1-h band power time-series in each frequency band (3,600 samples), using 3-min windows (180 samples) with 50% overlap, with a mean deletion performed on each segment. We then averaged the MSC spectrum the frequency range lower than 0.15 Hz to provide a single MSC estimate for correlations in infraslow envelopes of band power time-series for each contact pair.

Prior to applying these measures to icEEG data, we wanted to further characterize the MSC estimator. Importantly, the application of MSC, and indeed, all similar measures to neural time-series data necessitates the use of an estimator, as auto- and cross-spectra cannot be computed analytically. The most commonly used estimator for MSC is the fast Fourier transform (FFT)-based Welch’s overlapped segmentation approach (WOSA) (Carter et al., 1973a; Zaveri et al., 1999). In this method, the time-series are divided into overlapping segments. Using the FFT to estimate auto- and cross-spectra, the MSC is computed on each segment and averaged over the entire time-series. Formally the WOSA coherence estimator with *n* overlapping windows is given by:

$${\hat{\gamma }}_{\text{xy}}\left(\omega \right)=\frac{1}{n}\frac{\sum_{i=1}^{n}S_{\text{xy}}^{\left(i\right)}\left(\omega \right)}{\sqrt{\sum_{i=1}^{n}S_{\text{xx}}^{\left(i\right)}{\left(\omega \right)}^{2}\sum_{i=1}^{n}S_{\text{yy}}^{\left(i\right)}{\left(\omega \right)}^{2}}}$$

In the above, $S_{\text{xx}}^{\left(i\right)}\left(\omega \right)$ , $S_{\text{yy}}^{\left(i\right)}\left(\omega \right)$ , and $S_{\text{xy}}^{\left(i\right)}\left(\omega \right)$ are the auto- and cross-spectra of the *i*th windowed segments *x* ^*(i)*^ and *y* ^*(i)*^. MSC is then simply:

$$\text{MSC}={\left|{\hat{\gamma }}_{\text{xy}}\left(\omega \right)\right|}^{2}$$

Such estimators will have an associated bias and variance, where bias refers to the expected difference between the estimated value of a parameter and its true value, and variance refers to the spread of the sampling distribution after repeated distribution. Both must be characterized before applying these measures to the analysis of time-series data, primarily because establishing estimator bias and variance also allows us to set a threshold for considering an MSC value significantly nonzero (i.e. correlation not attributable to statistical noise). Descriptions of estimator performance for MSC estimation, including theoretical approximations of bias based on the analytic probability density function for the MSC estimate, were initially provided by Carter and colleagues (in the context of developing measures to estimate time delay between signals) (Carter et al., 1973a; Carter et al., 1973b; Carter, 1987). Characterization has also been performed specifically in the context of the icEEG (Zaveri et al., 1999). However, applying MSC to the measure of correlations in infraslow activity poses the challenge of balancing the tradeoff between overlapping windows that are sufficiently long to capture oscillatory activity in the infraslow range (thought to contain activity as slow as 0.01 Hz) and keeping windows short enough to provide a sufficient number of segments to minimize estimator bias and variance. This, combined with the fact that we computed MSC over band power time-series instead of on raw icEEG signals, suggested that further empirical characterization of the estimator in this context was necessary.

In a previous study, we used surrogate icEEG signals known to be uncorrelated (white Gaussian noise, randomized icEEG data, and pink noise) to determine the effect of frequency band, segment length, and signal power on estimator bias and variance. Of these surrogate signals, we found that white Gaussian noise did not faithfully recapitulate signal characteristics of icEEG data. In examining randomized icEEG data, we selected random pairs of electrodes from different patients (i.e. signals known to be uncorrelated), thereby allowing us to preserve the power spectrum and phase information of each of the signals. Finally, we generated pairs of pink noise signals, as the 1/*f* power spectrum matches well with what is expected of icEEG data. Briefly, we found that frequency band and signal power do not significantly affect MSC bias and variance, but increasing the segment length helps decrease bias and variance, with hour-long band power time-series being sufficiently long to bring bias and variance down to an acceptable level (Joshi et al., 2016). Interestingly, we also found that thresholds for considering infraslow envelope MSC significantly nonzero matched almost exactly between the randomized icEEG data and pink noise surrogates. In this study, we further characterize these effects and consider the effect of window size and window overlap on infraslow envelope MSC estimation using pink noise signals as surrogate data. The concordance in results between the methods above (pink noise surrogates and randomized icEEG data) in our prior work, along with the fact that using pink noise signals afforded us greater control over the parameters of surrogate signal generation, were the reasons for focusing on pink noise as our surrogate signal of choice for this study. Notably, these methods for empiric characterization of the coherence estimator using surrogate data are analogous to those used in other modalities, such as cardiovascular data (Faes et al., 2004).

We generated 5,000 pairs of pink noise signals that were 921,600 samples long (i.e., 1 hour of data sampled at 256 Hz). For each of these signals, we generated band power time-series by computing power spectral densities (PSDs) on a second-by-second basis. Power in each of the conventional EEG frequency bands defined above was computed by summing the PSD over the corresponding frequency range for each second. This gave us band power time-series at a 1-s resolution (i.e., 3,600 samples) for each signal. We then estimated infraslow envelope MSC between these time-series by computing the average MSC below 0.15 Hz between all these time-series. We first confirmed our previous results that there was no difference between estimator bias and variance between different frequency bands. Then, we used pairs of delta band power time-series to assess the effect of window size and window overlap.

Given that infraslow modulations can be as slow as 0.01 Hz, we decided that 3-min windows would be the shortest window size that could reliably pick up these fluctuations. To determine the effect of window size, we tested window sizes ranging from 1 to 20 min with 50% overlap. To assess the effect of window overlap, we fixed the window size to 3 min and varied the window overlap between 25%, 50%, and 75% overlap. We then examined the resulting distributions to optimize each of the parameters for the MSC estimation on real icEEG data.

### 2.4 Time lag analysis

We determined whether long-term correlation structure is preserved despite some time lag between the signals. Given that infraslow activity includes process on the order of many seconds to minutes, we expected some significantly nonzero infraslow envelope MSC to be preserved even if one of the signals was slightly shifted in time.

For each hour-long icEEG segment pre- and post-ASM taper, we selected 5,000 random electrode contact pairs and computed infraslow envelope MSC for the delta, theta, alpha, beta, and gamma bands. Using these same contact pairs, we then circularly shifted one signal of the pair by random amounts in 15 s increments, i.e. in a set of 5,000 pairs of signals, one signal of each pair was shifted by a random amount between 0 and 15 s, 15 and 30 s, etc., to give us 5,000 trials per patient for each time lag interval. We performed this analysis in 15 s increments through 300 s of lag. We then aggregated these data across all patients, giving us a total of 110,000 MSC values across all patients for each lag interval, and examined the resulting distributions to determine how the infraslow envelope MSC varies with time lag duration. We also determined whether and how the character of these distributions varied before and after ASM taper.

## 3 Results

### 3.1 Infraslow envelope magnitude-squared coherence estimator bias and variance

Based on our tests using 0 dB pink noise signals with infraslow envelope MSC computed using 3-min windows with 50% overlap, the mean and standard deviation of infraslow envelope MSC were 0.027 ± 0.007 in all frequency bands. The maximum MSC estimate observed over the 5,000 trials in any frequency band was 0.07. There was no significant difference in the MSC distributions across the different frequency bands.

**FIGURE 1:**
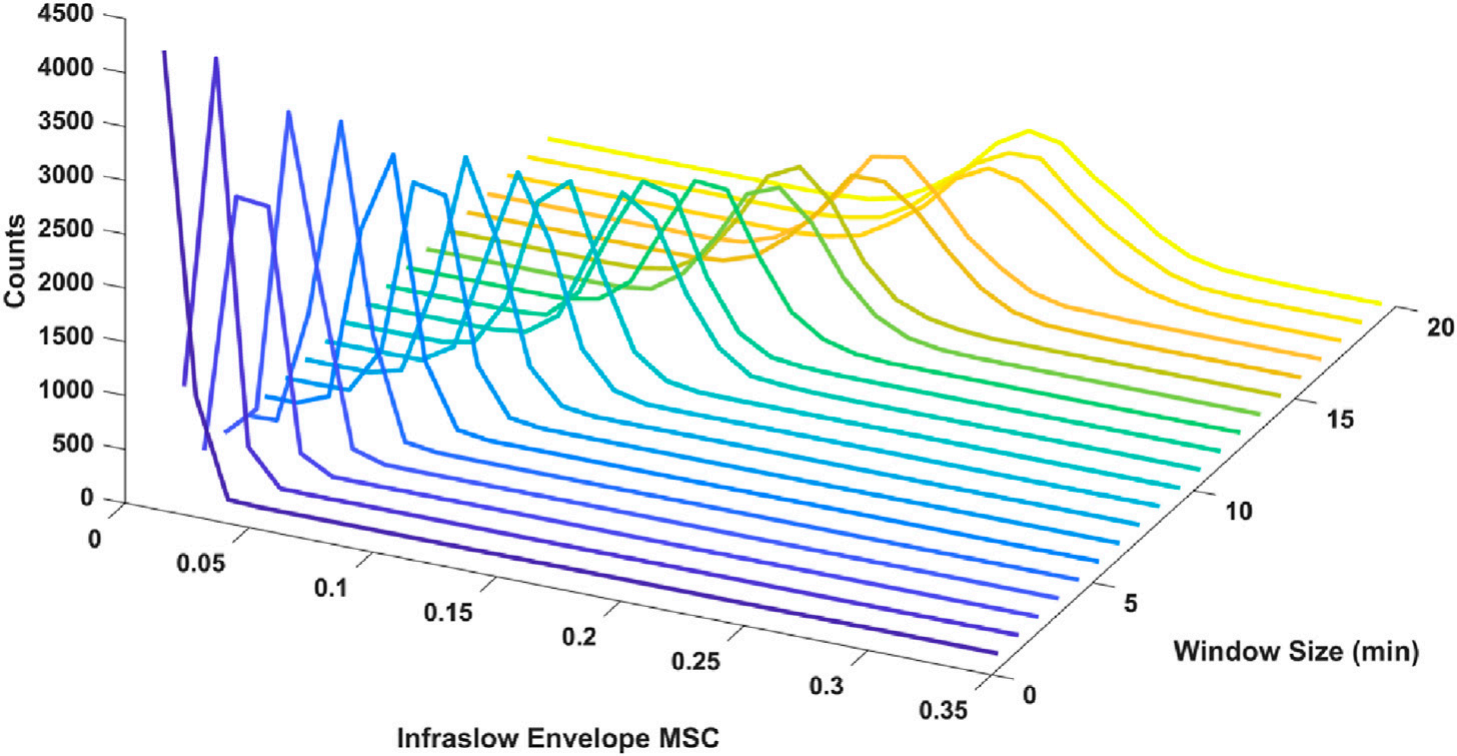
Histograms of infraslow envelope MSC estimates for 0 dB pink noise signals using different window sizes ranging from 1 to 20 min (each 5,000 trials). The mean and standard deviations for infraslow envelope MSC were 0.027 ± 0.007 for 3-min windows, 0.055 ± 0.011 for 6-min windows, 0.116 ± 0.021 for 12-min windows. Estimator bias and variance was minimized when 3-min windows were used.

We also tested the effect of window size in the MSC estimation on the resulting distribution of estimated infraslow envelope MSC values in the delta band only. Specifically, we examined window sizes ranging from 1 to 30 min with 50% overlap. There was a decrease in estimator bias and variance with smaller window size. For example, with 3-min, 6-min, 12-min windows, and 30-min windows with 50% overlap (20, 10, and 5 unique non-overlapping windows respectively), the mean and standard deviations for infraslow envelope MSC were 0.027 ± 0.007 for 3-min windows, 0.055 ± 0.0011 for 6-min windows, 0.116 ± 0.021 for 12-min windows, and 0.341 ± 0.048 for 30-min windows. The distributions are included in Figure 1. This also confirmed that a window size of 3 min would allow us to strike a balance between faithfully capturing fluctuations on the infraslow timescale while minimizing estimator bias and variance.

**FIGURE 2:**
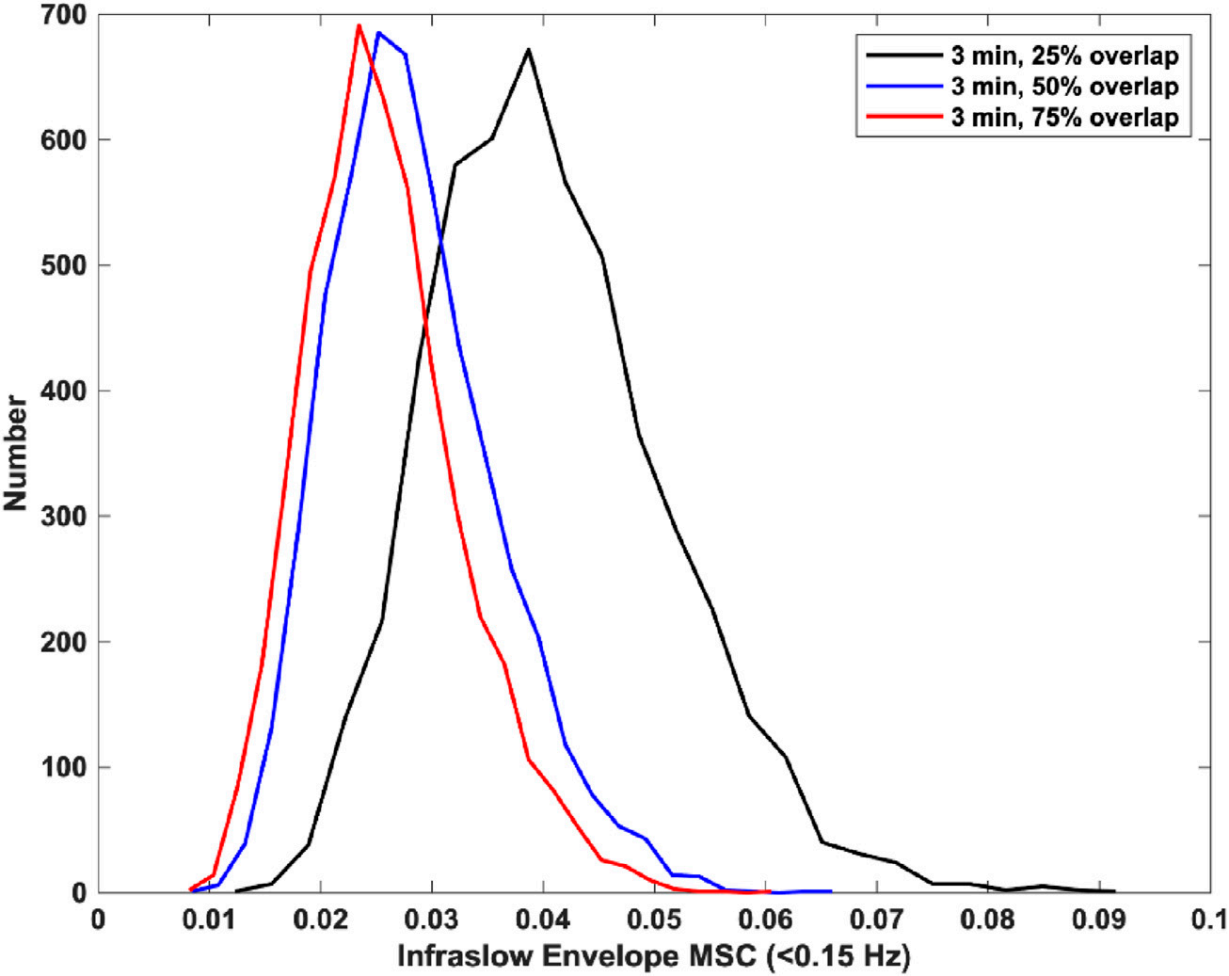
Histograms of infraslow envelope MSC estimates for 0 dB pink noise signals using 3-min windows with varying overlap (each 5,000 trials). The mean and standard deviations for infraslow envelope MSC estimates were 0.341 ± 0.039 for 25% overlap, 0.027 ± 0.008 for 50% overlap, and 0.025 ± 0.007 for 75% overlap.

Finally, we tested the effect of window overlap on the MSC estimation. Specifically, we used 3-min windows with 25% overlap, 50% overlap, and 75% overlap. There was a significant decrease in estimator bias and variance from 25% overlap to 50% overlap, and a non-significant decrease in bias and variance from 50% overlap to 75% overlap. The mean and standard deviations for infraslow envelope MSC estimates were 0.341 ± 0.039 for 25% overlap, 0.027 ± 0.008 for 50% overlap, and 0.025 ± 0.007 for 75% overlap. These distributions are provided in Figure 2. This indicated that increasing the window overlap beyond 50% would increase computational load without appreciably decreasing estimator bias and variance. We therefore used a window size of 3 min with 50% overlap for the icEEG analyses.

Based on the combination of these and our prior results, we were able to use 0.054 as a threshold for considering a mean infraslow envelope MSC value significantly nonzero (computed as the mean plus 3 standard deviations, p < 0.005) (Joshi et al., 2016).

### 3.2 Time lag analysis of intracranial EEG signals on- and off-ASM

**FIGURE 3:**
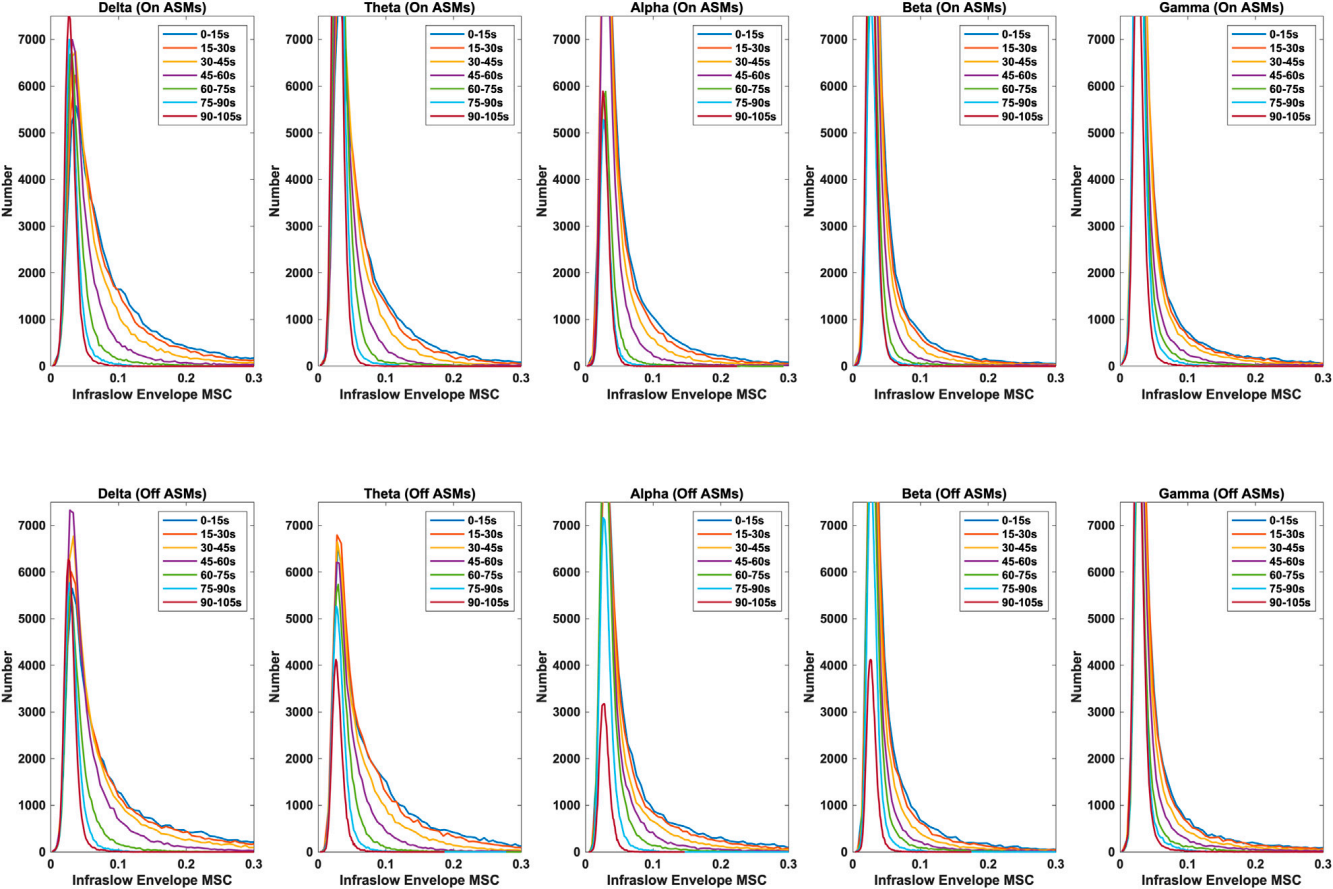
Distributions of infraslow envelope MSC with lags varying from 0 to 105 s for the delta, theta, alpha, beta, and gamma bands, both on- and off-ASMs. A strong positive tail in the distribution persists until about 105 s of lag. Infraslow envelope MSC is increased slightly, but significantly, after ASM taper (p < 0.01 based on Bonferonni correction, Wilcoxon rank sum test).

**FIGURE 4:**
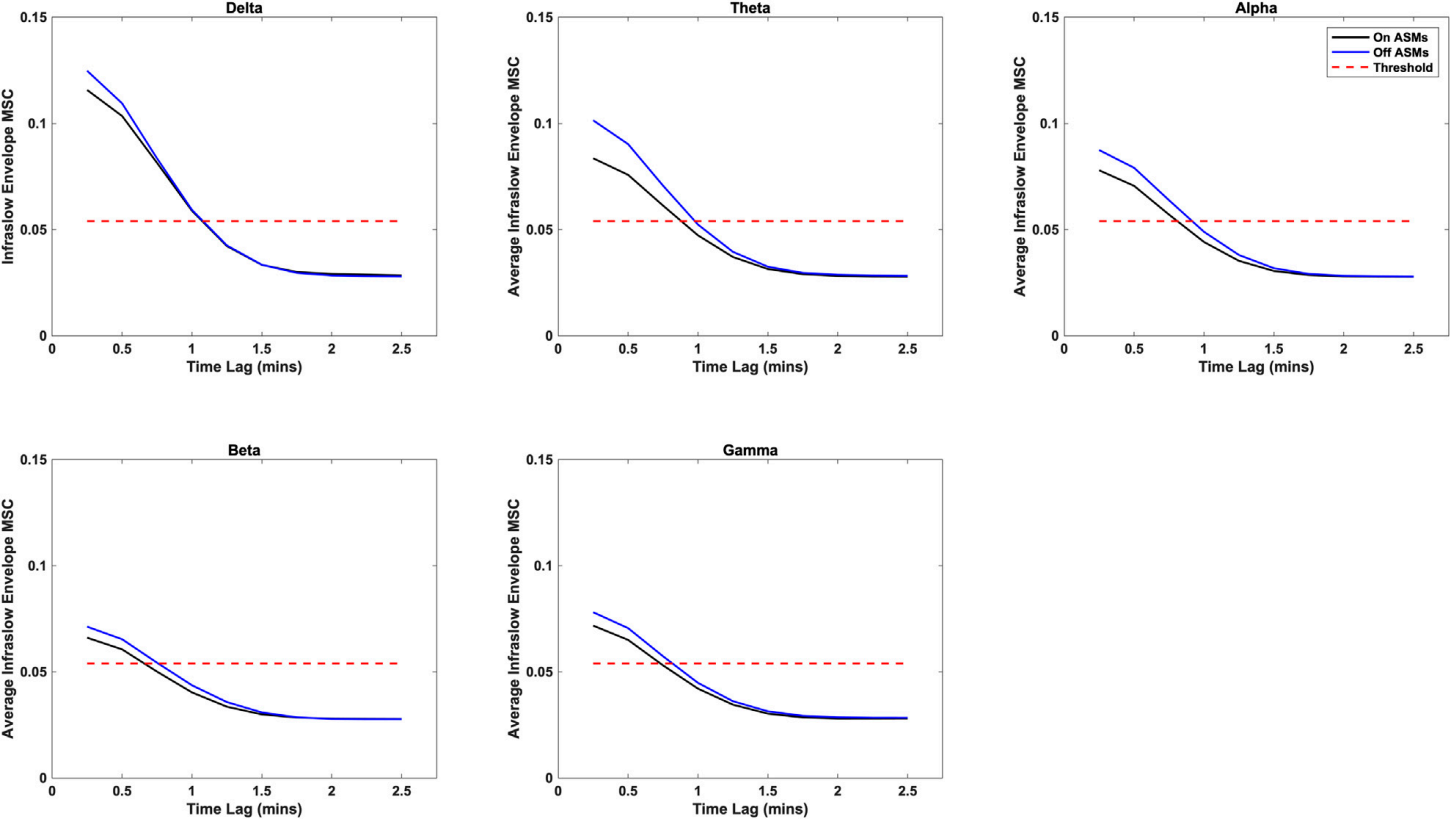
Average infraslow envelope MSC as a function of time lag for each of the frequency bands both on- and off-ASMs. The mean decreases monotonically with increasing lag. It drops below the statistical threshold of 0.054 at about 1 min of lag, and plateaus after 2 min of lag (full 300 s not pictured).

Time lag analysis showed that the infraslow envelope MSC distributions changed with increasing time lag. These distributions are provided in Figure 3. In particular, we observed a large positive tail in the MSC distributions that is present for time lags up to 105 s. These time lags were present in all frequency bands, and both before and after ASM taper. For time lags greater that 105 s, the MSC distributions matched those that were generated in surrogate testing of the MSC estimator. We also computed the mean infraslow envelope MSC as a function of time lag. These results are included in Figure 4. We found that the mean MSC decreases monotonically with increasing lag until about 105 s of lag, then plateaus until 300 s of lag. The average MSC remains above the threshold level of 0.054 until about 1 min of lag, though this depended on frequency band.

In comparing the pre- and post-ASM taper epochs, we found that although both exhibited similar results in relation to time lag (i.e. that the positive tail in the MSC distributions was present until about 105 s of lag), infraslow MSC increases slightly but significantly post-ASM taper epochs in all frequency bands (Wilcoxon rank-sum test, p < 0.01). These results are in agreement had observed previously in a smaller cohort of patients (Joshi et al., 2018).

## 4 Discussion

In this study, we sought to better characterize the timescale of infraslow activity captured by functional connectivity measures. We first examined the Welch’s overlapped segmentation approach of the estimation of magnitude-squared coherence as applied to the study of infraslow envelope correlations in the intracranial EEG. We found that signal power does not significantly affect estimator bias and variance. For an hour-long segment of data, estimator bias and variance were minimized when 3-min windows with 50% overlap were used when computing MSC. In the time lag analysis, we found that some significantly nonzero infraslow envelope MSC may be preserved until about 105 s of lag. Our study also shows a small but significant increase in MSC after ASM taper with preservation of results related to time lag across these two conditions.

In addition to these findings, consideration of bias and variance in the numerical estimation of functional connectivity measures is an important issue for network studies in neuroscience, but one that is often overlooked. In the specific case of infraslow envelope correlations and related slow activity, many prior studies do not provide benchmarking of measures used in the studies against surrogate data or similar methods (Nir et al., 2008; Brookes et al., 2011; Kucyi et al., 2018). This is important to consider, as observations of low, non-zero connectivity might reflect statistical noise in the estimation process, not true correlation in brain activity. In network neuroscience studies, therefore, it may not be enough to provide statistical analysis that differentiates between different brain regions or thresholding based on the distribution of connectivity values obtained from the data, as this will not account for problems with estimation of the measure itself. We also believe this highlights the utility of developing methods for generating representative surrogate data to benchmark measures prior to analyzing real data.

The results of the time lag analysis suggests that infraslow envelope correlations truly reflect modulations of higher frequency activity by ISOs, as previously described (Hughes et al., 2011; Mitra et al., 2018; Monto et al., 2008; Rodin and Funke, 2012; Watson, 2018) Prior work on ISOs indicates the lowest frequency oscillations observed in this range are around 0.01 Hz, which corresponds to a period of approximately 100 s (Aladjalova, 1957; Aladjalova, 1964; Hughes et al., 2011). This matches with our analysis, in which we observed positive tails in the MSC distribution until 90–105 s of lag. A limitation of this study was that ISOs cannot be measured directly from the original signal, as a highpass filter at 0.03 Hz is fixed in the hardware of our recording system to mitigate the effects of slow transients and electrode drift (Ebersole et al., 2014). However, these results indicate the feasibility of using infraslow envelope measures as a way of studying infraslow activity in the EEG in the absence of DC-coupled amplifiers.

The increase in MSC following ASM taper confirm the results of our prior work (Joshi et al., 2018). This is likely reflective of a general vulnerability to seizure that occurs post-taper, and we have previously observed a similar increase in infraslow envelope MSC in pre-seizure periods and during sleep (Joshi et al., 2018). The fact that nonzero MSC is preserved with increasing lag both before and after ASM taper suggests that ISOs are present in both states, though their expression may change depending on ASM load. Given that infraslow activity seems to modulate both cortical excitability and the occurrence of interictal spikes in epilepsy patients, further study of how infraslow activity is changed by ASM status is warranted (Vanhatalo et al., 2004). It is possible that infraslow envelope MSC may be a reflection of large-scale modulatory influences on smaller-scale processes and higher-frequency activity. Disruptions in this top-down modulation could increase vulnerability to seizures. Indeed, more recent work provides some evidence that phase-amplitude coupling between infraslow activity and high-frequency activity (>80 Hz) may have utility in distinguishing preictal and interictal states (Hashimoto et al., 2021). These combined results demonstrate the relevance of infraslow rhythms in clinical epilepsy in assessing seizure risk, and point toward their likely utility in novel methods of seizure forecasting.

## Data Availability

The datasets presented in this article are not readily available because the data used are prior clinical recordings from patients at Yale-New Haven Hospital. Requests to access the datasets should be directed to rasesh.joshi@childrens.harvard.edu.
